# In Vitro Assessment of the Physiologically Relevant Oral Bioaccessibility of Metallic Elements in Edible Herbs Using the Unified Bioaccessibility Protocol

**DOI:** 10.3390/molecules28145396

**Published:** 2023-07-14

**Authors:** Tatiana G. Choleva, Charikleia Tziasiou, Vasiliki Gouma, Athanasios G. Vlessidis, Dimosthenis L. Giokas

**Affiliations:** Department of Chemistry, University of Ioannina, 45110 Ioannina, Greece

**Keywords:** herbs, bioaccessibility, micronutrients, herbs, heavy metals, essential elements

## Abstract

In this work, the total content of seven metallic elements (Fe, Cu, Zn, Mg, Pb, Ni, and Co) in common edible herbs was determined and related to their bioaccessibility by an in vitro human digestion model. Specifically, the unified bioaccessibility protocol developed by the BioAccessibility Research Group of Europe (BARGE) was used to determine the release of each element during gastric and gastrointestinal digestion. The results show that Fe, Zn, and Mg are released during gastric digestion (34–57% Fe, 28–80% Zn, 79–95% Mg), but their overall bioaccessibility is reduced in the gastrointestinal tract (<30%). On the contrary, Cu is more bioaccessible during gastrointestinal digestion (38–60%). Pb, Ni, and Co exhibited similar bioaccessibility in both gastric and gastrointestinal fluids. Principle component analysis of the data shows that the classification of the nutritional value of herbs differs between the total and the gastrointestinal concentration, suggesting that the total concentration alone is not an adequate indicator for drawing secure conclusions concerning the nutritional benefits of edible plant species.

## 1. Introduction

Plants and plant-based products (such as vegetables, herbs, seeds, etc.) are among the most important food commodities in the diet of most countries and play a significant role in human nutrition. This is because they are available in large quantities and they are rich in a variety of important constituents, such as vitamins, polyphenols, antioxidants, metallic elements, proteins, etc., which are necessary for human nutrition and act protectively against serious clinical disorders and diseases [1]. In addition, many plant species contain pharmaceutically active compounds that have been used for centuries to treat diseases and improve human health [2]. The bioactive compounds in many plants have also been the basis for the design of functional foods and nutraceuticals that offer additional benefits to the human organism.

The composition of edible plants has been thoroughly investigated, and a vast amount of information is currently available for almost all known species. However, the presence of bioactive compounds in plants is not directly related to their uptake in the human organism. To obtain the benefits of these compounds, they must be released from the plant matrix and modified in the gastrointestinal tract to become bioaccessible and bioavailable [3]. Bioaccessibility refers to the amount of a compound that is released from the food matrix in the gastrointestinal tract and potentially becomes available for absorption, while bioavailability refers to the fraction of the compound that reaches systemic circulation and is utilized by the human organism [4]. Therefore, to evaluate the actual benefits of plant consumption, it is necessary to evaluate both the bioaccessibility and bioavailability of its active components.

Since the determination of bioavailability is an intricate and time-consuming procedure that requires in vivo tests and extensive clinical evaluation, bioaccessibility is most frequently tested, usually utilizing in vitro experimental procedures [5,6]. These procedures do not aim to accurately replicate the conditions found in the various compartments of the human digestive system, but they aim to simulate the physiological conditions and the main sequence of physicochemical events that occur during digestion. To accomplish this task, various components found in the mouth, the stomach, and the intestine, such as bile salts and enzymes (mucin, pepsin, pancreatin, etc.), organic molecules (such as glucose, urea, amino acids, etc.), and inorganic electrolytes, are used to mimic the composition of gastric and gastrointestinal fluids. Based on this rationale, several experimental protocols have been developed, such as the physiologically based extraction test (PBET), the simulation of the human intestinal microbial ecosystem (SHIME) procedure, the dynamic gastrointestinal model (TIM), and the in vitro gastrointestinal method, among others [3,4,7]. These methods, although designed to simulate the human digestive tract, exhibit many dissimilarities that do not enable a direct data comparison [8]. To standardize operational procedures and harmonize biomimetic extraction tests, the BioAccessibility Research Group of Europe (BARGE), developed the standardized unified BARGE method (UBM), using surrogate digestive fluids, (saliva, gastric juice, duodenal juice, and bile), for which their chemical composition is adjusted to be similar to that of human physiology [9]. The experimental procedure of the UBM method involves two sequential extraction steps: gastric digestion, in which the saliva and the gastric juice are added to the solid substrate (mouth and stomach compartments), followed by gastrointestinal digestion, in which the duodenal fluid and the bile salts are also included along with the gastric fluid. This harmonized method, although initially developed to evaluate the risks associated with the unintentional ingestion of soil, has been also applied to several food commodities, such as seeds [10,11], nutritional supplements [12], seaweeds [13], and recently roots [14], fruits, and vegetables [15].

Among foods of plant origin, fresh herbs are commonly used as food seasonings in small quantities. However, they are physically, biochemically, and nutritionally similar to vegetables and contain a variety of bioactive phytochemicals, such as vitamins, terpenoids, flavonoids, phenolic acids, and essential mineral elements [16,17]. However, due to the low amount that is consumed compared with other green leafy vegetables, studies on the bioaccessibility of their bioactive components in the gastrointestinal environment are scant. Among the most important bioactive components found in plant and herb species are metallic elements, which play an important role in various biological functions. For example, elements such as Fe, Cu, Zn, and Mg are involved in enzymatic reactions, the transport of oxygen, the functioning of nerve and muscle cells, the formation of DNA, etc., and their deficiency is associated with major clinical disorders and diseases. However, apart from essential elements, plant species may also contain heavy metal ions as a result of contamination during cultivation, transportation, and storage. These metals may accumulate in the human organism and cause adverse health effects. Although some of these heavy metals (e.g., Ni, Co, Mn) are involved in biochemical functions at low concentration levels, they exhibit negative effects at higher concentration levels. On the other hand, heavy metals such as Pb, As, Cr, etc., pose significant risks to human health [18]. In this regard, this work aims to investigate the nutritional value of fresh herbs through the determination of the gastric and intestinal bioaccessible fractions of four essential elements (Fe, Mg, Zn, and Cu) using the validated UBM method. Moreover, the presence and bioaccessibility of heavy metal ions (Pb, Ni, and Co) are also assessed to evaluate the role of fresh herbs in the uptake of heavy metal ions. Importantly, since edible herbs belong to the same species worldwide, evaluating the bioaccessibility of metallic elements can serve as a foundation for assessing their contribution to the uptake of these elements.

## 2. Results and Discussion

### 2.1. Total Content of Essential Elements and Heavy Metals

The total content of the examined metal ions was first determined to get insight into the quality of herbs. In addition, the total concentration of metal ions was used for mass balance calculations that were necessary for the quality control of the experimental data. Table 1 summarizes the results (average from three independent sampling campaigns) from the analysis of seven metal ions in the selected herbs. The concentration of metal ions changed according to the order: Mg (454–922.5 mg/Kg) > Fe (19.4–156.2 mg/Kg) > Zn (16.8–138.9 mg/Kg) > Cu (0.3–6.1 mg/Kg) > Ni (0–6.3 mg/Kg) > Co (0–0.3 mg/Kg) > Pb (0–1 mg/Kg). The total content of metal ions was within the range of values reported in other studies [19,20,21]. Interestingly, the concentration of Fe in the samples was reversibly related to that of Zn in most herbs, which may be attributed to the cultivation conditions. Fe is related to respiration activities, N fixation, and electron transfer [22], and increases with photosynthetic activity while Zn competes with the ligands that promote Fe accumulation, reducing Fe transport to the aerial parts of the plants when photosynthetic activity is lower [23]. Since Fe in parsley and oregano is lower than Zn, we can conclude that Fe is less accumulated, indicating a possible lower photosynthetic activity. For parsley, this can also be related to the fact it has been grown in mixed cultivations, where agronomic conditions are adjusted to the needs of all species and not to specific species.

**Figure 1:**
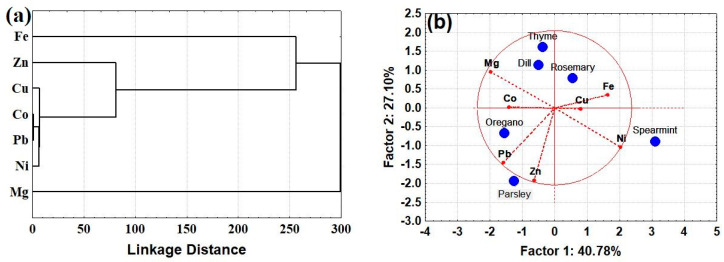
(**a**) Hierarchal cluster analysis (single linkage Euclidean distances) between metal ions. (**b**) Graphic of scores (blue) and loadings (red) of PCA in the evaluation of the contents of Cu, Fe, Mg, Zn, Cu, Co, Pb, and Ni in edible herbs. The raw data (total concentration of metal ions in the herbs) were used for the classifications.

To better visualize the results, a cluster analysis plot was built up using the raw data (i.e., total metal content in the plant) as shown in Figure 1a. Metal ions such as Fe, Mg, Zn, and Cu (which are all essential elements) form separate clusters from Ni, Co, and Pb, indicating their different sources, while Cu is grouped between these species, indicating that part of Cu might also stem from the agricultural activity. A similar observation can also be made for Mg, which is grouped separately from all metallic elements. Utilizing PC1 × PC2, ca. 70% of the data variance was explained (Figure 1b), allowing for a partial discriminatory classification of the herbs in four categories: (1) parsley and oregano, depending on the concentrations of Pb and Zn; (2) spearmint, which shows a large dependence on the concentrations of Ni; (3) dill and thyme based mainly on the concentrations of Mg and Co; and (4) rosemary, which seems to have a different profile in Fe and Cu.

### 2.2. Bioaccessibility of Metallic Elements

The above results confirm the importance of herbs as a source of metallic elements in human nutrition. However, as previously discussed, the total content of metal ions in each herb does not reflect the actual benefits or risks associated with its consumption. Therefore, the in vitro UBM digestion protocol was used to estimate the human bioaccessibility of nutrients and toxic metal ions from herb consumption. The bioaccessible fraction was defined as the ratio between the content of a metal ion in the bioaccessible fraction and the total metal content in the plant [12] as follows:

BF(%) = (bioaccessible content)/(total content) × 100.

**Figure 2:**
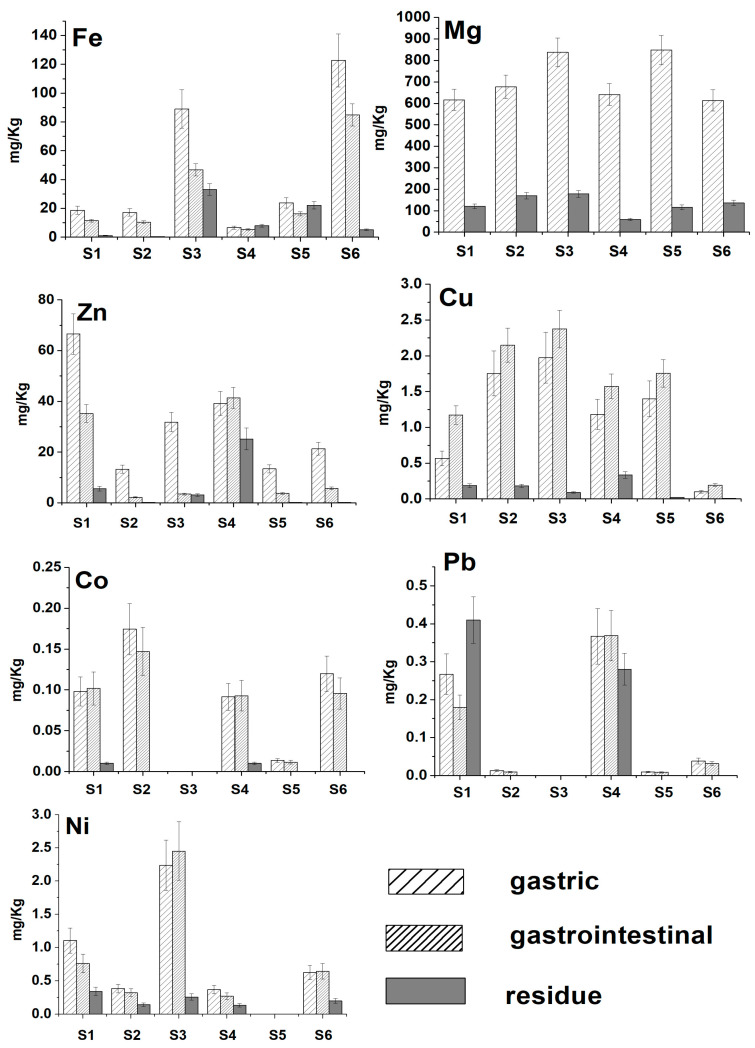
Gastric, gastrointestinal, and residual concentrations of essential elements and toxic metal ions in herbs. S1: Parsley; S2: Dill; S3: Spearmint; S4: Oregano; S5: Thyme; S6: Rosemary.

The results for bioaccessible and residual fractions in herbs following gastric and gastrointestinal digestion are depicted in Figure 2. The recovery of metal ions, determined from mass balance calculations (bioaccessible and residual fraction compared with the total content), yielded satisfactory results that ranged from 70–122% for Fe, Cu, Zn, and Mg and 62–113% for Pb, Ni, and Co, indicating the acceptable accuracy of the overall experimental procedure. The bioaccessible fractions for each metal ion are depicted in the bar plots of Supplementary Materials: Figure S1. From these plots, it can be seen that the bioaccessibility of Fe, Mg, Zn, and Ni was higher in the gastric phase, while Cu exhibited higher bioaccessibility in the gastrointestinal phase. On the other hand, the bioaccessibility of Pb and Co was comparable in the gastric and gastrointestinal phases. It should be noted that the concentration of Mg in the gastrointestinal phase was not determined due to the high concentration of Mg salts that were added to simulate the composition of the gastrointestinal tract.

The bioaccessibility of metal ions in the gastric phase can be mainly attributed to the low pH, which increases the solubility of metals, as well as to the presence of pepsin, which is more effective in acid conditions to break down proteins [24,25]. The lower bioaccessibility of Fe and Zn in the gastrointestinal phase may be due to the higher pH of the duodenal fluid (approximately 6.3), which may cause the formation of insoluble Fe and Zn oxyhydroxide complexes and hamper their intestinal adsorption [11,26]. Moreover, the presence of fibers, oxalates, tannins, polyphenols, and mainly phytates (which is a main source of phosphorous) plays a significant role in the reduced bioaccessibility of metal ions in the gastrointestinal phase by complexing metal ions or forming metal precipitates that reduce their bioaccessibility [15,26,27,28,29]. In contrast to other elements, Cu bioaccessibility was higher in the gastrointestinal phase. This is attributed to the fact that Cu, due to its unpaired electrons, forms stable complexes with proteins that also include pepsin [30,31], which is the main enzyme during gastric digestion. On the other hand, the increase in the bioaccessibility of Cu in the gastrointestinal fluid is in agreement with previous reports and can be attributed to the almost neutral pH and the presence of low-molecular-weight organic acids (e.g., ascorbic, malic, etc.), which have been associated with the improved bioaccessibility of Cu in the gastrointestinal tract [11,30,32,33].

Regarding toxic metal ions, Pb exhibited similar bioaccessibility in the gastric and gastrointestinal phases. This can be attributed to the fact that pepsin in the gastric fluid may not be able to release most of Pb, but pancreatin in the duodenal fluid can decompose cell walls and increase the release of Pb into intestinal juice [34]. However, the action of pancreatin in the gastrointestinal tract competes with the coprecipitation of Pb by iron oxides, which restricts the bioaccessibility of Pb [14].

The presence of Co in the samples is attributed to exogenous sources (i.e., human activities) since these herbs do not naturally contain cobalamin. The low pH and the presence of pepsin in the gastric fluid releases Co, which exhibits a high affinity for proteins and amino acids [35]. The high bioaccessibility of Co in the gastrointestinal phase may be ascribed to the formation of low-molecular-mass cobalt complexes [36] or the formation of chloride complexes (due to the presence of high concentrations of chloride salts), which exhibit high solubility in both gastric and gastrointestinal fluids [37]. Finally, the bioaccessibility of Ni in the gastric and gastrointestinal phases was similar to that reported in previous studies [19]. The bioaccessible concentration of Ni in the gastric phase is slightly higher than in the gastrointestinal phase, possibly due to Ni coprecipitation and adsorption on Fe oxides at neutral pH (6.3–7.0) of the duodenal fluid [38]. However, the chelation of Ni by some enzymes, such as pepsin, bile, and mucin, may inhibit Ni adsorption by Fe oxides in the intestine and maintain part of Ni as bioaccessible [38].

**Figure 3:**
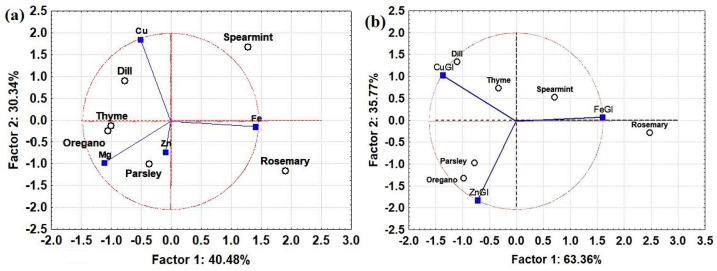
Classification of herbs by comparing element bioaccessibility and total concentration: (**a**) the total concentration of four essential elements and (**b**) the bioaccessible (gastrointestinal) concentration of four essential elements. The open black circles represent the graphic scores and the blue squares represent the factor loadings.

Using the total concentration of elements and their concentration in the gastrointestinal fluid, PCA was used to identify potential similarities between different herbs in terms of element bioaccessibility. This could be used to design functional products with improved bioaccessibility of essential elements. The score plots in Figure 3 show that the total concentrations and the concentrations in the bioaccessible fraction do not lead to similar classifications, which means that the total concentration does not provide a representative evaluation of the bioaccessibility of elements. Based on the results of Figure 3b, oregano and parsley exhibit the highest bioaccessibility of Zn while dill and thyme offer improved bioaccessibility of Cu. Finally, spearmint seems to offer improved bioaccessibility of Fe. However, spearmint was found to have the highest total concentration of Cu. Therefore, although the bioaccessibility of Cu in the gastrointestinal fluid (38.9%) was lower than thyme (≈45.2%), it still offered a high content of bioaccessible Cu in terms of absolute concentrations (ca. 2.38 mg/Kg in spearmint and 1.75 mg/Kg in thyme). Similarly, although only 30% of Zn in oregano was bioaccessible in the gastrointestinal tract, the total concentration was high, contributing to its high bioaccessibility. Hence, both the total concentration of elements and their bioaccessibility in the gastrointestinal tract should be evaluated to identify potential herb synergies and design functional foods that maximize the bioaccessibility of metallic elements.

## 3. Materials and Methods

### 3.1. Reagents and Solutions

All reagents (salts and organic chemicals) were at least of analytical grade and purchased from major suppliers. Ultrapure grade HNO_3_ and H_2_O_2_ for inorganic trace analysis were a product of Supelco. Alpha amylase (*Bacillus* sp. ≥ 1500 units/mg protein, Merck, Darmstand, Germany), mucin from porcine stomach (type II, Sigma-Aldrich, Steinheim, Germany), pepsin from porcine gastric mucosa (≥500 U/mg, Merck, Darmstand, Germany), bovine serum albumin (Sigma Aldrich, Steinheim, Germany), lipase from porcine pancreas (>150 units/mg protein, Sigma-Aldrich, Steinheim, Germany), pancreatin from porcine pancreas (350 FIP-U/g protease, 6000 FIP-U/g lipase, 7500 FIP-U/g amylase, Merck, Darmstand, Germany), and bile from porcine pancreas (Sigma-Aldrich, Steinheim, Germany) were used to simulate the enzyme composition of gastric and gastrointestinal fluids. The multielement standard solution 6 for ICP (Supelco TraceCERT^®^, 100 mg/ L, Sigma-Aldrich, Steinheim, Germany) was used to prepare standard metal ion solutions for calibration. Before use, all glassware was rinsed with acetone and water, soaked in 2 M ultrapure HNO_3_ overnight, thoroughly washed with distilled water (<2 μS/cm), and dried in a ventilated oven.

The preparation of the digestive fluids used in the UBM test (saliva, gastric, duodenal, and bile) was performed the day before use. Each fluid consisted of a mixture of an inorganic electrolyte solution, a solution of organic compounds, and a solution containing the appropriate enzymes [9]. The composition of each fluid is compiled in Table S1. Each solution was prepared separately, mixed, and stirred for 4 h. The pH of each solution was then recorded and, if necessary, adjusted with the dropwise addition of NaOH or HCl, as follows: salivary fluid 6.5 ± 0.5, gastric fluid 1.1 ± 0.1, duodenal fluid 7.4 ± 0.2, and bile 8 ± 0.2. Before the application of the UBM protocol, all fluids were incubated at 37 °C for 1 h.

### 3.2. Instrumentation

An ICP OES (Shimadzu, Kyoto, Japan, ICPE-9800), equipped with a semiconductor CCD detector, was used for the measurements. The readouts were recorded with axial view mode, at an exposure time of 30 s. The plasma torch was operated with an RF power and frequency of 1.2 kW and 27 MHz, respectively, a coolant argon flow rate of 10 L/min, an auxiliary argon flow rate of 0.6 L/min^−1^, and a carrier flow rate of 1.0 mL/min, with simultaneous recording of analytical signals at wavelengths of 239.349, 238.204, and 259.940 nm for Fe, 213.598, 224.700 and 324.754 nm for Cu, 202.548, 206.200, 213.856 nm for Zn, 279.553, 280.270 and 285.213 nm for Mg, 228.616, 237.862 and 238.892 nm for Co, 221.647, 231.604 and 341.476 nm for Ni, and 216.999, 220.353, and 405.783 nm for Pb as a quality control measures.

### 3.3. Samples

Samples of fresh herbs (except for oregano that was obtained air-dried) were purchased from the same suppliers in three independent sampling campaigns. All herbs were cultivated in organized cultivations in different parts of Greece and were nonorganic. Some species (parsley, spearmint, and dill) were reported to be grown in mixed cultivations with other species, but no specific information was given.

### 3.4. Sample Preparation and Determination of Metal Ions

Fresh herbs were manually cut into small pieces using a ceramic knife, macerated in liquid nitrogen, and lyophilized for 72 h in a benchtop freeze dryer (Alpha 1-2 LD Plus) (Christ, Osterode am Harz, Germany). The dry solids were ground to powder with a ball mill and stored in a desiccator until analysis and for no longer than 3 days. For determination of the total concentration of metal ions, 0.15 ± 0.02 g of the dry (lyophilized) material was weighted and transferred to PFA microwave digestion vessels (Savillex, Eden Prairie, MN, USA). Then 1 mL of ultrapure concentrated HNO_3_ and 0.6 mL of ultrapure H_2_O_2_ (30%, *w*/*w*) were added. The solution was predigested at 50 °C for 30 min to decompose organic matter and release gases. After cooling, the vessels were tightly sealed and exposed to microwave-assisted extraction. Specifically, in the first step, power was held at 430 W for 5 min to aid the mineralization of the organic components. Then, energy was increased to 720 W for 4 min, and decomposition was completed by exposure at 900 W for 5 min, twice. The vessels were cooled in a water bath, and the extracts were transferred to volumetric flasks by filtering through Whatman No. 40 (ashless, metal-free) filters using dilute ultrapure HNO_3_ (2 M).

For the determination of metal ions calibration plots were prepared by analyzing standard solutions in the range of 20–500 μg/L. For each element, the part of the calibration plot that enabled the best linear range was used. Then, the sample solutions were analyzed either directly or after appropriate dilution with 2 M ultrapure HNO_3_.

### 3.5. In Vitro Simulation of Gastrointestinal Digestion

For each solid sample, the UBM test was performed in triplicate, including one blank sample (without solid) as control. Therefore, for every herb sample, 8 liquid samples were generated (3 replicates of the gastric phase and 1 gastric fluid blank, and 3 replicates of the gastro-intestinal phase plus 1 gastro-intestinal fluid blank). First, 0.8–1.0 g of samples was weighed in polypropylene flasks (50 mL), and 4.5 mL of salivary solution was added followed by manual agitation for 10 s. Then, 6.75 mL of gastric fluid was added, and the pH was adjusted to 1.2 ± 0.05, using small volumes of concentrated HCl. The mixture was then incubated for 60 min at 37 °C in a RotoFlex Plus end-over-end rotator (Agros, Chicago, IL, USA) at 40 rpm. The pH of the gastric phase was measured to verify that it is lower than 1.5. The gastric samples were centrifuged at 4500 rpm for 15 min, and the supernatant solution was carefully retrieved with a glass pastier pipette and acidified with HNO_3_ to stop the enzymatic reactions and preserve the samples until analysis.

For the gastrointestinal digestion phase, 13.5 mL of duodenal and 4.5 mL of biliary fluids were added to the gastric samples, and the pH was adjusted to 6.3 ± 0.4 with 5 mol/L NaOH. The flasks were mixed in the end-over-end rotator for 4 h at 37 °C at 40 rpm, centrifuged at 4500 g for 15 min, and the supernatant was acidified with HNO_3_. All samples were sequentially filtered through Whatman No 40 and Nylon filters of 0.45 µm pore size. All filters were also rinsed with 2 M HNO_3_ to avoid metal sorption.

The solid material (residual fraction after digestion) was dried in an oven and decomposed by microwave irradiation using the same procedure that was used for the extraction of total metal content from the raw (lyophilized) herb samples.

### 3.6. Principle Components and Cluster Analysis

Principle component analysis (PCA) was used to unravel relationships between the profile of metallic elements and herb species, as well as their bioaccessibility. We used the herb species as a grouping variable and the concentration of metal ions (total, gastric, and gastrointestinal) as variables for the analysis and classification of the herbs. Cluster analysis (CA) was also used to identify relationships between the metal ions as expressed by their concentration levels. Two sets of data were used: the first consisted of the raw data containing the total concentration of metallic elements in the herbs, while the second set was the concentration of metallic elements determined in the gastric and gastrointestinal extracts.

## 4. Conclusions

The assessment of the elemental composition of common edible herbs shows that they constitute a supplementary source of metallic elements in the human body and that they may also contribute to the uptake of heavy metals. The bioaccessible (gastric and gastrointestinal) concentrations of essential (Fe, Zn, Mg, and Cu) investigated by the UBM method show that the most bioaccessible element is Cu, followed by Fe and Zn. Moreover, metal ions, such as Pb, Ni, and Co, have been found to be readily bioaccessible and potentially bioavailable in the gastrointestinal tract. Therefore, although the concentration of metals may not be high, special attention must be given to the cultivation conditions and processing of herbs to minimize contamination from heavy metals. The results from bioaccessibility testing also show the potential for designing functional foods and nutraceuticals with improved bioaccessibility of essential elements.

## Figures and Tables

**Table 1:** The total content of metallic elements in mg/Kg (average results from triplicate measurements in three independent sampling campaigns).

	Fe	Mg	Zn	Cu	Co	Pb	Ni
Parsley	35.9 ± 5.2	719.7 ± 88	111.4 ± 17	2.4 ± 0.8	0.2 ± 0.1	0.9 ± 0.1	2.0 ± 0.08
Dill	39.8 ± 4.7	768.2 ± 101	17.6 ± 2.6	5.4 ± 0.8	0.3 ± 0.1	<LOD	0.8 ± 0.08
Spearmint	156.2 ± 25	454.0 ± 76	46.7 ± 5.5	6.1 ± 1.1	<LOD	<LOD	6.3 ± 0.9
Oregano	19.4 ± 3.8	830.1 ± 91	138.9 ± 21	3.9 ± 1.0	0.2 ± 0.1	1.0 ± 0.2	0.8 ± 0.1
Thyme	58.1 ± 5.4	922.5 ± 98	16.8 ± 3.3	3.9 ± 0.8	<LOD	<LOD	<LOD
Rosemary	236.0 ± 27	682.1 ± 84	28.4 ± 4.2	0.3 ± 0.1	0.2 ± 0.1	0.1 ± 0.1	1.6 ± 0.5

<LOD: Lower than the detection limit.

## Data Availability

The authors confirm that the data supporting the findings of this study are available within the article and its supplementary materials.

## Supplementary Materials

The following supporting information can be downloaded at: https://www.mdpi.com/article/10.3390/molecules28145396/s1, Table S1: Composition of the digestive fluids used in the in vitro UBM bioaccessibility method. Concentrations are in g/L unless otherwise stated. Figure S1: Recovery (%) of essential elements and toxic metal ions in herbs. Patterns inside the bar blots show the bioaccessible fraction (BF, %) in the gastric and gastrointestinal phases and the residual amount remaining after digestion.
