# Supplementary material for: In Vitro Assessment of the Physiologically Relevant Oral Bioaccessibility of Metallic Elements in Edible Herbs Using the Unified Bioaccessibility Protocol
Source: Molecules. 2023 Jul 14;28(14):5396. doi: 10.3390/molecules28145396 (PMC10386267; doi:10.3390/molecules28145396)
Supplement: Supplementary file 1 [file molecules-28-05396-s001.zip › molecules-2422911-supplementary.pdf]

SUPPORTING INFORMATION

In Vitro Assessment of the Physiologically Relevant Oral Bioaccessibility of Metallic Elements in Edible Herbs Using the Unified Bioaccessibility Protocol

Tatiana G. Choleva, Charikleia Tziasiou, Vasiliki Gouma, Athanasios Vlessidis,

Dimosthenis L. Giokas

Department of Chemistry, University of Ioannina, 45110, Ioannina, Greece

Table S1. Composition of the digestive fluids used in the *in-vitro* UBM bioaccessibility method. Concentrations are in g/L unless otherwise stated.

		Salivary fluid	Gastric fluid	Duodenal fluid	Bile
**Inorganic salts**	KCl	1.8	1.6	1.13	0.8
	NaCl	0.6	5.5	14.0	10.5
	NH_4Cl	-	0.6	-	-
	$CaCl_2$	-	0.8	0.4	0.4
	$MgCl_2$	-	-	0.1	-
	NaH_2PO_4	1.8	0.5	-	-
	KH_2PO_4	-	-	0.16	-
	KSCN	0.4	-	-	-
	$NaHCO_3$	3.4	-	6.7	11.6
	$NaSO_4$	1.1	-	-	-
	HCl (% w/v)	-	1.3	0.04	0.03
**Organic compounds**	Urea	0.4	0.2	0.2	0.5
	Uric acid	0.03	-	-	-
	Glucose	-	1.3	-	-
	Glucuronic acid	-	0.04	-	-
	Glucosamine hydrochloride	-	0.7	-	-
**Enzymes**	α-Amylase	0.5	-	-	-
	Mucin	0.05	6.0	-	-
	BSA	-	2.0	2.0	3.6
	Pepsine	-	5.0	-	-
	Pancreatin	-	-	18.0	-
	Lipase	-	-	3.0	-
	Bile	-	-	-	6.0

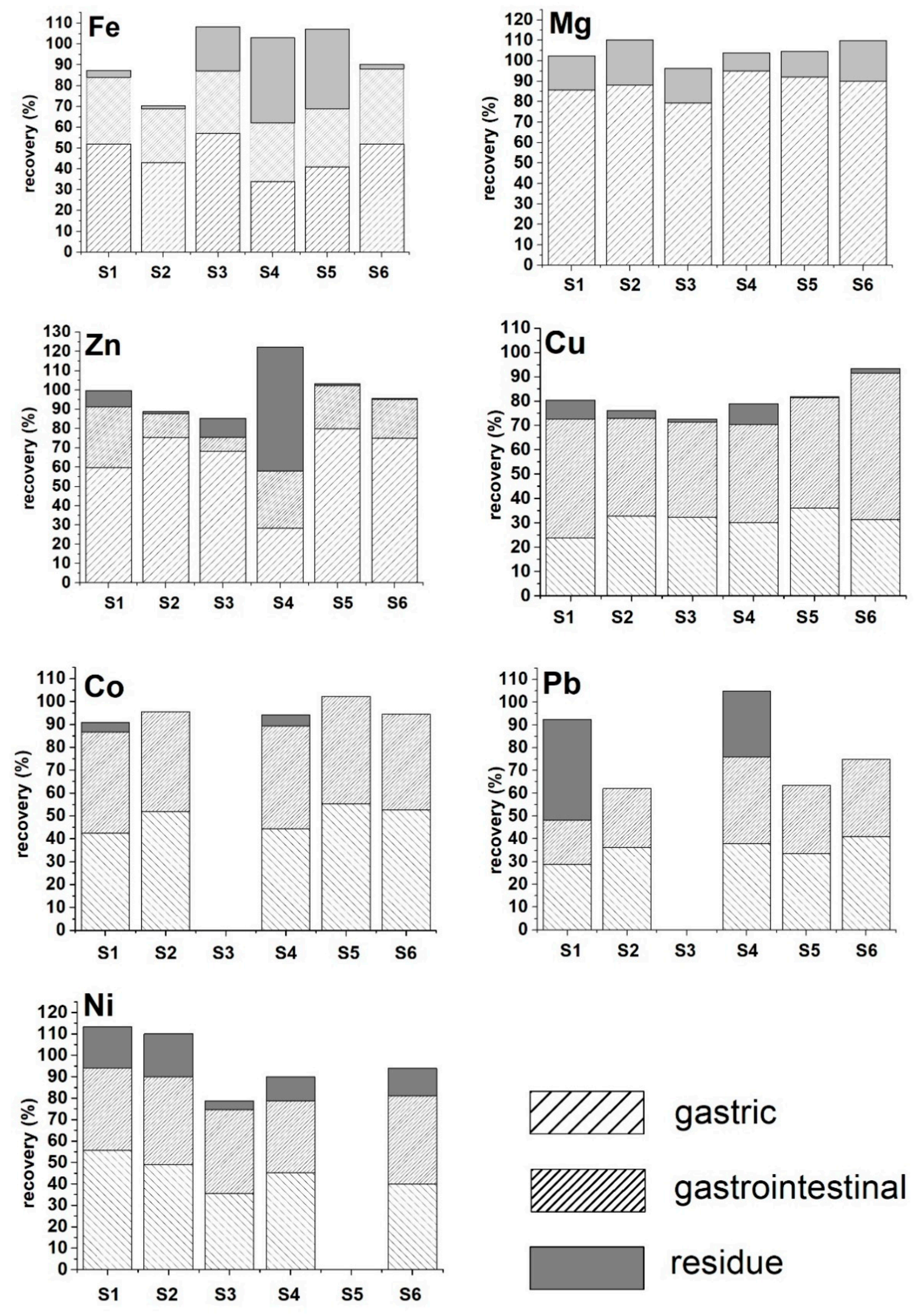

Figure S1. Recovery (%) of essential elements and toxic metal ions in herbs. Patterns inside the bar blots show bioaccessible fraction (BF, %) in the gastric and gastro-intestinal phases and the residual amount remaining after digestion. S1: Parsley, S2: Dill, S3: Spearmint, S4: Oregano, S5: Thyme, S6: Rosemary.
